# Impact of stocking densities on growth, organ index, serum biochemistry, gut morphology and microbiota of young ducks in a rice-duck-crayfish coculture system

**DOI:** 10.5713/ab.24.0488

**Published:** 2025-01-24

**Authors:** Xiao Long He, Zhen Hua Liang, Ze Heng Huang, Lian Bing Qi, Yan Wu, Jia Liu, Tao Huang, Jing Bo Liu, Jin Song Pi, Hao Zhang

**Affiliations:** 1 Institute of Animal Husbandry and Veterinary Science, Hubei Academy of Agricultural Sciences, Wuhan, China; 2 School of Life Science and Engineering, Southwest University of Science and Technology, Mianyang, China; 3 College of Animal Science and Technology, Yangtze University, Jingzhou, China

**Keywords:** Duck, Growth, Intestinal Microbiota, Rice-Duck-Crayfish Coculture System, Stocking Density

## Abstract

**Objective:**

The rice-duck-crayfish (RDC) coculture system, an ecologically efficient breeding strategy that accommodates natural behavior of ducks and improves their welfare. The optimal stocking density and its impact on duck health in this system remains undetermined. The study examined the impact of stocking densities on growth, organ index, serum biochemistry, gut morphology and microbiota of ducks in RDC system.

**Methods:**

A total of five hundred and forty 20-day-old Nonghu No. 2 ducks were randomly allocated based on density: low-density (LD; 8 birds/666.67 m^2^), medium-density (MD; 12 birds/666.67 m^2^) and high-density (HD; 16 birds/666.67 m^2^) groups, with three replicates in each group, and the symbiosis period was up to 40 days until rice tasselling.

**Results:**

There were no significant differences in final body weight, average daily gain, or feed:gain ratio between groups (p>0.05); however, the liver and spleen indices of ducks in HD group were significantly greater than those in LD group (p<0.05). The serum albumin concentration in HD group decreased, whereas creatine kinase activity increased (p<0.05). Additionally, the ileal crypt depth significantly increased and the ileal villus height and villus/crypt ratio significantly decreased in ducks in MD and HD groups compared to LD group (p<0.05). Moreover, the abundance of cecal *Deferribacterota* and *Spirochaetota* increased significantly (p<0.05), while the abundance of *Firmicutes* decreased significantly (p<0.05) with increasing stocking density. Moreover, the increase in stocking density significantly decreased the abundance of some beneficial bacteria (*Faecalibacterium* and *Fournierella*) and increased the abundance of some harmful bacteria (*Mucispirillum* and *Brachyspira*) (p<0.05).

**Conclusion:**

These results suggest that moderately HD breeding doesn’t significantly affect duck growth, but increased stocking density led to changes in cecal microbiota and dysbiosis. Reducing stocking density positively affects immune parameters and ileum morphology. However, due to the limited number of total replicates of the study, further research is needed to validate the reliability of the results.

## INTRODUCTION

China is the world’s largest producer and consumer of duck products [1]. Ducks in the rearing process using a variety of breeding modes, in which the rice-duck ecological farming system, as the organic combination of planting and aquaculture rearing mode, has been emphasized and popularized by some countries and regions in recent years [2]. The rice-duck-crayfish (RDC) co-culture system is a comprehensive and efficient integrated farming model that integrates rice cultivation, duck rearing and crayfish farming. In this system, the daily activities and foraging behaviors of ducks and crayfish achieve natural control of weeds and pests in the rice field, effectively replacing the use of pesticides and herbicides in conventional planting patterns and improving the ecological environment of the rice field [3]. At the same time, duck and crayfish feces can be used as organic fertilizers to promote the propagation of aquatic plants in the paddy field, and can be used as food for ducks and crayfish, forming a multilevel cycle of ecological farming system [4]. The RDC co-culture system not only fully releases the natural habits of ducks, but also facilitates the brood and rearing of young ducks, which is an ideal way to practice the concept of animal welfare and realize ecologically friendly aquaculture.

The stocking density plays an important role in influencing animal production and breeding. A stocking density that is too high can negatively affect the health, immune status and growth and development of poultry [5]. Conversely, a stocking density that is too low will result in poor weeding and pest control, waste of resources, and reduced economic benefits [6]. A study reported that ducks were stocked at densities of 40 m^2^/bird, 50 m^2^/bird, and 60 m^2^/bird, and it was found that lower stocking densities resulted in greater average body weights at capture [7]. And increased stocking density (75/ha to 300/ha) in a rice-duck system delayed the first laying period and decreased egg production [8]. In addition, an increase in stocking density affects the composition and diversity of the gut microbiota, which in turn affects the overall health and productivity of ducks [9]. Studies have shown that changes in stocking density affect the composition and diversity of soil microflora under an ecological farming model [10]. Studies have also observed changes in the characterization of the intestinal flora of crayfish in paddy farming environments [11]. However, there are limited specific studies on duck gut microbiota in RDC systems. In the ecological farming system, rice, ducks and crayfish are mutualistic and intercropping, and optimizing duck stocking density plays an important role in the growth of rice and ducks as well as the corresponding economic and ecological benefits. But the optimal stocking density and group size in this system remain unclear, especially concerning the growth and breeding effect of young ducks raised in rice field.

Nonghu No. 2 laying duck is a famous laying duck synthetic line in Hubei Province (China), which was validated by China Genetic Resources Validation Committee in 2021 and included in Animal Genetic Resources in China: Poultry. It is well-known for the high reproductive performance and has stronger resistance to adversity than other breeds. This experiment used the “Nonghu No. 2 Young Duck as the duck species in the RDC system. To investigate the changes of the young ducks’ growth performance, body size, serum biochemistry, intestinal development, and intestinal bacterial flora in different stocking density, and to clarify the appropriate density of Nonghu 2 young ducks in the RDC system and the effects on the ducks.

## MATERIALS AND METHODS

### Ethics statement

The authors state that they have complied with the ethical policies outlined on the journal’s author guidelines page and have obtained the necessary approval from the relevant ethical review committee. The experimental methods were carried out in accordance with the Guidelines for Experimental Animals established by the Ministry of Science and Technology. All animal-related procedures received approval from the Animal Ethics Committee of Hubei Academy of Agricultural Sciences (2021-620-000-001-019). Additionally, the authors affirm adherence to EU standards for the protection of animals used in scientific research.

### Experimental design and bird management

The experiment selected nine plots of high standard farmland, each with 3,333.35 m^2^. Three treatment groups with different densities were set up: 8 birds/666.67 m^2^ (low-density [LD]), 12 birds/666.67 m^2^ (medium-density [MD]), 16 birds/666.67 m^2^ (high-density [HD]), with three replicates in each group, and each plot was treated as a replicate. A total of five hundred and forty 20-day-old female Nonghu 2 young ducks (mean body weight was 223.76±1.48 g) were randomly divided into three treatment groups according to different stocking densities. The duck were provided by the Poultry Experimental Base of the Hubei Academy of Agricultural Sciences (Wuhan, China). During the test period, the ducks in each group were fed complete diet, and grazing + quantitative supplemental feeding was adopted, with supplemental feeding at 07:00 am and 17:00 pm. every day. The amount of supplemental feeding was adjusted according to the changes in duck age, with 47 g/duckling fed for 20 days, after which the amount of feeding was increased by 2 g/day/duck. The diet composition and nutrient levels are detailed in Table 1. The nutrient composition of feed ingredients and the metabolic energy of the diet are calculated with reference to the National Standard of the People’s Republic of China “Nutrient Requirements of Laying Ducks” (GB/T 41189-2021), based on the proportion of feed ingredients. The nutritional composition of the feed was calculated and analyzed using nutritional value data for each ingredient based on national standards. A ditch 2 m wide and 1 m deep was dug around the field, nylon nets were installed around the field to prevent the escape of ducks and crayfish as well as the invasion of hostile organisms, and duck hutches were established for the ducks to rest and feed at night. After ploughing the farmland, crayfish fry (20 kg/666.7 m^2^) were placed in the ditches. Approximately 15 days after transplanting the seedlings, 20-day-old ducklings were introduced into the field, and the ducks were conditioned with musical signals to establish conditioned reflexes for feeding management. Musical signals were used to recall ducks from each field to the duck hutches for feeding before each feeding. Ducks were similarly recalled to the duck hutches at the end of the experiment for fasting sampling. No pesticides, fertilizers or herbicides were sprayed on the rice during the symbiosis stage, and the ducks were driven out of the field when the rice was tasselled and grouted to end the symbiosis, which lasted for 40 days. During the 40-day period, the ducks were exposed to natural daylight during the day. At night, we provided continuous artificial lighting at 5 lux to deter predators (such as weasels). The test site was the Shishou city Shuangshui Shuanglv production base in Hubei Province (Shishou, China).

### Growth performance and body development measurement

The weights of the ducks were measured as they entered and exited the paddy field to calculate the average daily gain (ADG). The daily feed intake of each replicate was recorded, which is calculated by subtracting the residual feed from the feeding amount. However, there was no residual feed in the supplemental feed trough during the entire experimental period. Therefore, We determined the average daily feed intake (ADFI) by the total feed intake of each replicate of ducks per day and the number of days of feeding during the entire experimental period. At the end of the experiment, two ducks were randomly selected per replicate (n = 6 per group) to collect samples. The body size indices of ducks were determined after 12 h of fasting according to the methods described in Nomenclature and Metric Calculation Methods for Poultry Production Performance (NY/T823-2020). Specifically, body oblique length refers to the distance from the shoulder joint to the ipsilateral sciatic tuberosity measured along the body surface with a tape measure. The keel length was the distance from the anterior end of the keel eminence to the end of the keel and was measured with a tape measure along the body surface. The tibial length was the distance from the supratarsal joint to the third and fourth toes and was measured in a straight line with a Vernier calliper. The tibial girth was the circumference of the middle of the tibia (tarsometatarsal) and was measured with a tape measure. The half-diving length was measured with a tape measure from the tip of the rostrum to the midpoint of the line connecting the two acetabular tuberosities.

### Organ indices and intestinal morphology

After the live weights were obtained, the ducks were euthanized by injecting air into the heart. Later, the ducks were eviscerated and the heart, liver, spleen, pancreas, gizzard and proventriculus were removed from the ducks to calculate the organ indices. The organ index (g/kg) was calculated as organ weight (g)/body weight (kg). From the gizzard to the beginning of the mesentery as the duodenum, the jejunum and ileum are distinguished by the position of Meckel’s diverticulum, and a 2cm section of duodenum, jejunum and ileum was also removed and fixed in 4% formaldehyde solution after gently rinsing the contents with saline. The fixed intestinal segments were dehydrated in ethanol, embedded in paraffin, sectioned and stained with haematoxylin-eosin, and observed morphologically under a microscope (Nikon ECLIPSE E100; Nikon, Tokyo, Japan). The six intact intestinal villi were selected to measure the villus height (VH), crypt depth (CD) by using image processing software (ImageJ 1.8.0, National Institute of Health, Bethesda, MD), and the villus height/crypt depth ratio (VH/CD) was calculated.

### Serum biochemical parameters

At the end of the experiment, blood samples were collected from six ducks in each group using a vacuum blood collection tube, with the samples obtained from the wing vein. After blood collection, the blood was allowed to stand for 2 h, and then centrifuged at 1,100×g for 10 min after blood coagulation. Subsequently, the serum was collected and transferred to 1.5 mL centrifuge tubes, and stored at −80°C for further use. The serum concentration of total bilirubin (TBIL), glucose, triglyceride, total cholesterol, high-density lipoprotein, low-density lipoprotein, alkaline phosphatase, aspartate aminotransferase, alanine aminotransferase, total protein (TP), albumin (ALB), immunoglobulin G, creatine kinase (CK), and cholinesterase were measured using a commercial kit (Chongqing Runkang Biotechnology Co., Ltd., Chongqing, China), with a fully automated biochemical analyzer (PUZS-300X, Beijing Plan New Technology Co., Ltd., Beijing, China). The concentration of globulin was the difference between total protein and ALB [12].

### 16S rRNA sequencing

Duck cecal contents were collected using sterile freezing tubes, flash-frozen in liquid nitrogen and then transferred to a −80°C freezer to be stored for testing. Afterwards, high-throughput sequencing was performed on the cecal samples of each group. Genomic DNA was extracted from the samples using the CTAB method, and after testing for concentration and purity. Polymerase chain reaction (PCR) amplification of the 16S rRNA V3–V4 region was performed using DNA as a template and the V3–V4 universal primer sequences 341F (5′-CCTAYGGGRBGCASCAG-3′) and 806R (5′-GGACTA CNNGGGGTATCTAAT-3). The PCR products were detected by 2% agarose gel electrophoresis followed by magnetic bead purification, enzyme labeling for quantification, and establishment of sequencing libraries. The libraries were quantified by Qubit and Q-PCR, and then sequenced using NovaSeq 6000 for PE250 upsequencing. The data of each sample were split from the downstream data based on the barcode sequence and PCR amplification primer sequence. The barcode and primer sequences were truncated, and the data were spliced, quality controlled and removed chimeric sequence using FLASH (version 1.2.11) and fastp software (version 0.23.1) to obtain valid data. The initial amplicon sequence variants (ASVs) and feature tables were obtained by noise reduction using DADA2 or deblurring in QIIME2 software (version QIIME2-202202). The ASVs were further analysed for species annotation using QIIME2 software to obtain species abundance at different taxonomic levels. Alpha diversity indices were calculated and weighted and unweighted based distance matrices were computed using QIIME2 software and β diversity analysis was performed by R software (4.0.3). Biomarkers of microbiota differences were analysed using linear discriminant analysis effect size (LEfSe) software (1.1.01), and microbial metagenomic function prediction was performed using PICRUSt.

### Statistical analysis

The SAS 9.4 software (SAS Institute, Cary, NC, USA) was used to perform one-way analysis of variance (ANOVA) on the experimental data, with Tukey’s test used to determine the significant differences between the mean values of treatment. The experimental unit for statistical analysis was each replicate plot. Polynomial contrasts for linear and quadratic responses were used to evaluate the effects of increasing stocking density on duck performance. Microbiota that changed significantly in the cecum microbiota phylum and genus level were analyzed by T-test test. All differences were considered significant at p<0.05. The data are expressed as the mean and standard errors of the mean. Data visualization was performed via GraphPad Prism 9.5.0 software.

## RESULTS

### Effects of stocking densities on the growth performance of young ducks in rice-duck-crayfish system

As shown in Table 2, there was no significant effect of different stocking densities on the final body weight, ADG or the F: G ratio of the ducks in the RDC coculture system (p>0.05). In addition, the half-diving length of the ducks in the HD group was significantly greater (p<0.05) than that in the LD group, but the body oblique length, keel length, tibial length, tibia girth and tibia weight were not significantly different (p>0.05) among the different stocking density groups (Table 3).

### Effects of stocking densities on the organ indices of young ducks in rice-duck-crayfish system

As shown in Table 4, the liver and spleen indices were significantly (p<0.05) greater in ducks in the HD group than in those in the LD group. However, the heart, pancreas, gizzard and proventriculus indices did not significantly differ (p>0.05) among the different stocking density groups.

### Effects of stocking densities on the serum biochemistry of young ducks in rice-duck-crayfish system

As shown in Table 5, compared with those in the LD group, the serum ALB concentration decreased and the CK activity increased significantly in the HD group (p<0.05). Additionally, compared with the LD group, there was a trend of lower serum TP concentration in the HD group (p = 0.072), while the TBIL concentration showed a trend of increase (p = 0.084). However, there was no significant difference in the other indices among the groups (p>0.05).

### Effects of stocking densities on the intestinal morphology of young ducks in rice-duck-crayfish system

As shown in Table 6, there was no significant difference (p>0.05) between the VH, CD and VH/CD of duodenum and jejunum between different stocking density. However, the ileal VH was significantly lower in the HD group than in the LD group (p<0.05). Moreover, the ileal CD was significantly greater in the MD and HD groups than in the LD group (p<0.01), while the VH/CD of ducks in the MD and HD groups was significantly lower than that in the LD group (p<0.01). Ileal VH and VH/CD decreased linearly with increasing density (p<0.05) and ileal CD increased linearly and quadratically (p<0.05).

### Effects of stocking densities on the cecal microbiota diversity of young ducks in rice-duck-crayfish system

**Figure 1:**
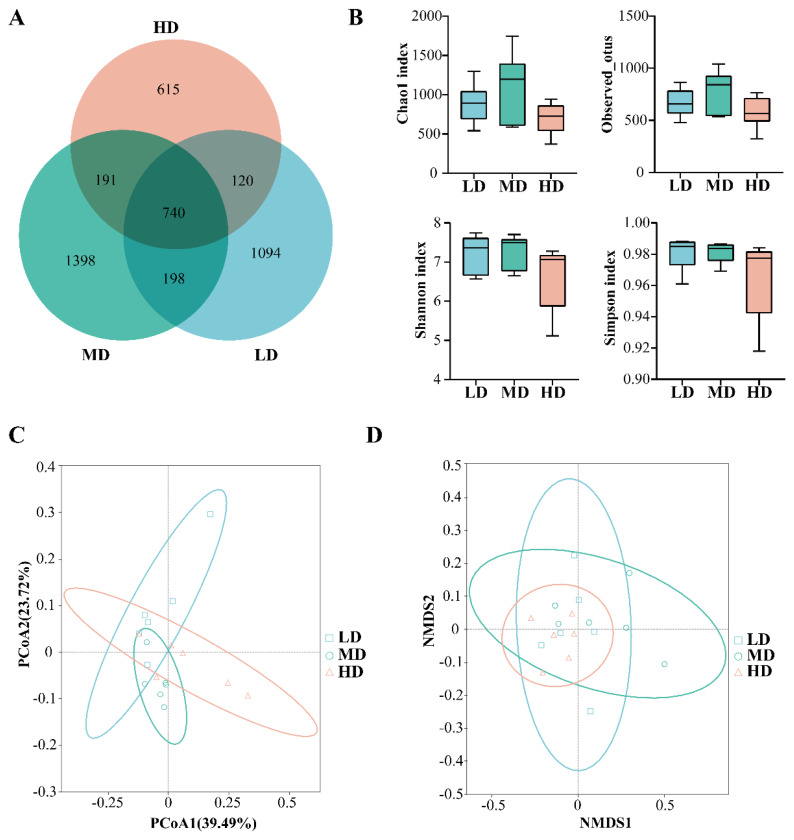
Effects of stocking densities on the diversity of cecal microbiota in RDC system. (A) Venn diagram of the shared or unique ASVs of LD, MD and HD groups. (B) The chao1 index, shannon index, simpson index, and observed_otus was used to describe the α diversity of gut microbiota of ducks. (C) Principal coordinate analysis (PCoA) based on weighted UniFrac distance of all the samples. (D) Non-metric multidimensional scaling analysis (NMDS) (n = 6 per group). LD, 8 birds/666.67 m^2^; MD, 12 birds/666.67 m^2^; HD, 16 birds/666.67 m^2^. HD, high-density; MD, medium-density; LD, low-density; RDC, rice-duck-crayfish; ASV, amplicon sequence variants.

In the cecum microbiome study, a total of 1,856,300 raw sequences were obtained by sequencing, and a total of 1,335,380 valid reads were obtained after quality control and filtering. We obtained the total number of ASVs between each group by sequence cluster analysis. The Venn diagram shows the number of ASVs that were shared or unique among the different densities. A total of 4,356 ASVs were obtained between the three groups, with 1,094 ASVs unique to the LD group, 1,398 ASVs unique to the MD group, and 615 ASVs unique to the HD group, with a decrease in the number of ASVs in the HD group (Figure 1A).

The effects of different stocking densities on the microbial diversity of duck cecal were further analysed. The α diversity results showed that there was no significant difference in the diversity indices among the different stocking density groups (Figure 1B). In addition, principal coordinate analysis (PCoA) and nonmetric multidimensional scaling (NMDS) were used to compare the β diversity of the caecal microbiota, and PCoA1 and PCoA2 explained 39.49% and 23.72% of the total data variance, respectively. The results showed a significant separation of clusters between groups with different stocking densities (Figures 1C, 1D). This result indicated that increased stocking density affected the cecal microbiota flora of ducks.

### Effects of stocking densities on the cecal microbiota composition of young ducks in rice-duck-crayfish system

**Figure 2:**
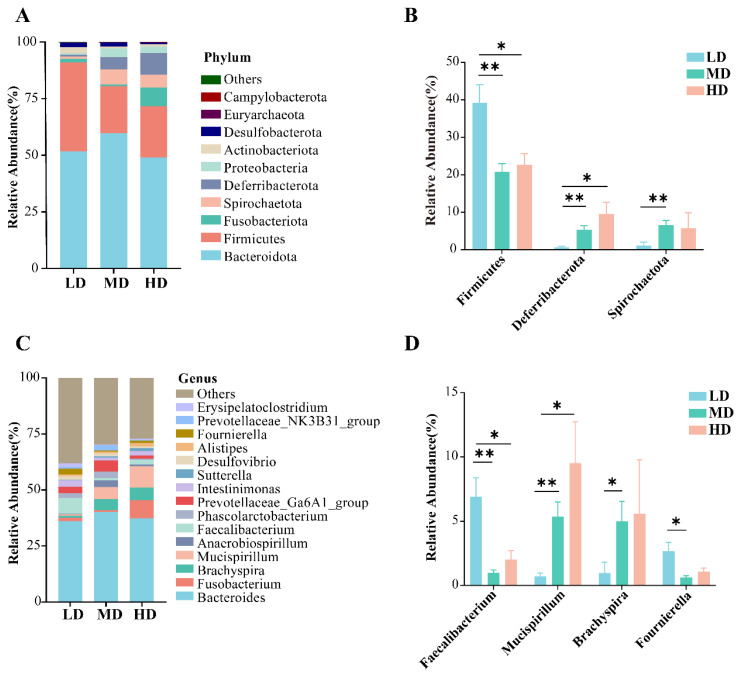
Effects of stocking densities on the composition of cecal microbiota in RDC system. (A, C) Changes in the relative abundance of cecal microbiota at levels of phylum and genus, respectively. (B, D) Differential bacterial relative abundance at the phylum and genus level between groups. Data are expressed as mean ± SEM (n = 6 per group). Asterisks indicate significant differences (* p<0.05, ** p<0.01). LD, 8 birds/666.67 m^2^; MD, 12 birds/666.67 m^2^; HD, 16 birds/666.67 m^2^. LD, low-density; MD, medium-density; HD, high-density; RDC, rice-duck-crayfish; SEM, standard error of the mean.

We further determined the composition of the duck cecal microbiota at the phylum and genus levels. At the phylum classification level, the abundance of the top 10 species were analysed, and the results showed that *Bacteroidota*, *Firmicutes, Fusobacteriota, Spirochaetota, Deferribacterota, Proteobacteria, Actinobacteriota, Desulfobacterota, Euryarchaeota, and Campylobacterota* were the major phyla in the cecal microbial community, accounting for 99% of the total phyla (Figure 2A). Among them, *Bacteroidota* and *Firmicutes* were the dominant phyla in the cecal of the ducks in the LD, MD and HD groups, accounting for 91.07%, 80.53% and 71.74% of the total phyla, respectively. The relative abundance of *Firmicute*s was significantly lower (p<0.05) in the MD and HD groups than in the LD group. However, the relative abundance of *Deferribacterota* and *Spirochaetota* significantly increased in the MD group compared to the LD groups (p<0.01). Moreover, the relative abundance of *Deferribacterota* was significantly greater (p<0.05) in the HD group than in the LD group (Figure 2B). At the genus level, we analysed the first 15 species and showed that *Bacteroides* was the dominant genus in the three groups accounting for approximately 40% of the total, while the remaining genera were *Fusobacterium, Brachyspira, Mucispirillum, Anaerobiospirillum, Faecalibacterium* and others (Figure 2C). The relative abundance of *Faecalibacterium* was significantly lower in the MD and HD groups than in the LD group (p<0.05), while that of *Fournierella* was significantly lower in the MD group than in the LD group (p<0.05). The relative abundance of *Mucispirillum* significantly increased (p<0.05) in the MD and HD groups, while that of *Brachyspira* significantly increased (p<0.05) in the MD group (Figure 2D).

**Figure 3:**
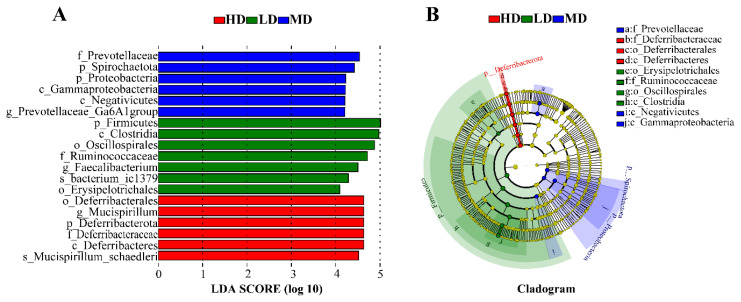
The linear discriminant analysis effect size (LEfSe) analysis of cecum differential microbiota between three groups. (A) Linear discriminant analysis (LDA) bar showed the change of the abundance of each species on the difference between the three groups (LDA score>4.0). (B) The cladogram shows the phylogenetic distribution of microbiota between three groups. LD, 8 birds/666.67 m^2^; MD, 12 birds/666.67 m^2^; HD, 16 birds/666.67 m^2^. HD, high-density; MD, medium-density; LD, low-density.

The biomarkers that were significantly different among the three groups were further analysed using LEfSe (linear discriminant analysis (LDA) score > 4), as shown in Figure 3, and a total of 19 dominant microorganisms at different taxonomic levels were found: 7, 6, and 6 in the LD, MD, and HD groups, respectively. The marker species in the LD group were mainly *p__Firmicutes, c__Clostridia, o__Oscillospirales, o__Erysipelotrichales, f__Ruminococcaceae, g__Faecalibacterium*, and *s__Bacterium_ic1379*. The marker species in the MD group were *p__Spirochaetota, p__Proteobacteria, c__Gammaproteobacteria, c__Negativicutes, f__Prevotellaceae*, and *g__Prevotellaceae_Ga6A1_*. The marker species in the HD group were *p__Deferribacterota, c__Deferribacteres, o__Deferribacterales, f__Deferribacteraceae, g__Mucispirillum*, and *s__ Mucispirillum_schaedleri* (Figures 3A, 3B).

### Functional prediction of the cecal microbiota

**Figure 4:**
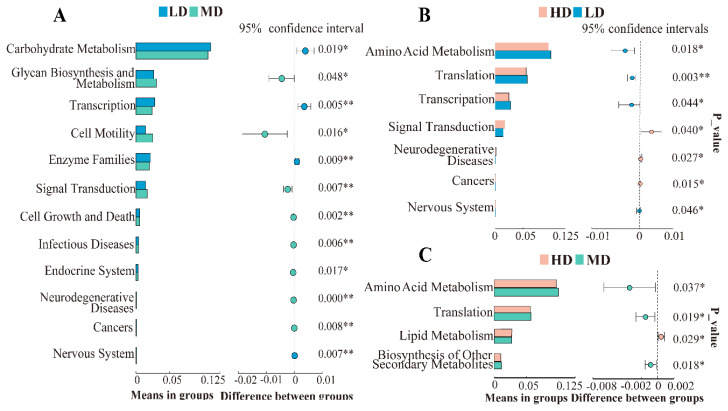
Differences in the KEGG pathway at level 2 of the cecal microbiota. (A) The metabolic pathway of gut microbiota with significant differences between LD group and MD group. (B) The metabolic pathway of gut microbiota with significant differences between HD group and LD group. (C) The metabolic pathway of gut microbiota with significant differences between HD group and MD group. Asterisks indicate significant differences (* p<0.05, ** p<0.01). LD, 8 birds/666.67 m^2^; MD, 12 birds/666.67 m^2^; HD, 16 birds/666.67 m^2^. LD, low-density; MD, medium-density; HD, high-density; KEGG, Kyoto encyclopedia of genes and genomes.

To predict functional changes in microorganisms in the cecum, we analysed the possible levels of the Kyoto encyclopedia of genes and genomes (KEGG) pathways by PICRUSt. The functional differences between groups with different stocking densities at the 2-level pathway level are shown in Figure 4. The abundance of functional genes involved in carbohydrate metabolism, transcription (genetic information processing), and enzyme families was significantly lower in the MD group than in the LD group (p<0.05). The abundance of functional genes involved in glycan biosynthesis and metabolism, cell motility, signal transduction (environmental information processing), cell growth and death, and infectious diseases significantly increased in the MD group (p<0.05) (Figure 4A). The abundance of functional genes involved in amino acid metabolism, translation and transcription (genetic information processing) was significantly lower in the HD group than in the LD group (p<0.05), while the abundance of genes involved in signal transduction was significantly greater (p<0.05) (Figure 4B). In addition, the abundance of functional genes involved in lipid metabolism was significantly greater (p<0.05), and the abundance of genes involved in amino acid metabolism was significantly lower (p<0.05) in the HD group than in the MD group (Figure 4C).

## DISCUSSION

As an ecological breeding mode, the RDC coculture system not only improves the utilization efficiency of resources, but also improves the rearing environment and welfare of animals, and promotes their natural growth; however, the duck stocking density under the RDC coculture system impacts the whole ecosystem. An increase in stocking density will have a negative impact on the growth performance of animals, in particular, a high stocking density is not conducive to animal welfare and health [5,13]. However, there are inconsistencies in the results of numerous studies on the effects of stocking density on poultry growth performance. Studies have shown that an increase in stocking density from 14 to 20 birds/m^2^ resulted in a significant decrease in broiler body weight, ADG and relative growth rate with increasing age, as well as a decrease in feed intake and an increase in the F: G ratio [14]. A study showed in geese that an increase in stocking density from 15 to 35 birds/m^2^ significantly decreased ADG and average daily feed intake, but did not affect F: G [15]. And the final body weight and weight gain were significantly reduced by increasing stocking densities (13 kg/m^2^ to 21 kg/m^2^ vs. 5 kg/m^2^ to 9 kg/m^2^) in both ducklings and growing ducks, but there was no significant effect on the F: G ratio in either period [16]. However, some studies reported no significant effect of different stocking densities (5, 10, 15 or 20 birds/m^2^) on the ADG or ADFI of broilers [17]. There was no significant difference in the body weight, weight gain, or the F: G ratio among 42-day-old broilers at 30, 35, or 40 kg/m^2^ [18]. The results of this experiment showed that different stocking densities had no significant effect on the body weight, ADG or F:G ratio of ducks. This finding is similar to that of a previous study. Some studies have shown that the decline in growth performance due to increased stocking density is related to reduced feed intake [19]. In this study, the use of fixed quantity feed and the RDC coculture system for autonomous foraging may have reduced the adverse effects of stocking density on growth. Moreover, the natural ecological environment of the paddy field may have varying effects on individual ducks, as this spatial environment could lead ducks to exhibit different levels of activity and behavioral patterns.

Body size indices is are important indicators of growth and developmental traits in poultry. A study on 7 to 49-day-old Hyline Brown laying hens revealed that tibial length, keel length and breast width decreased with increasing stocking density [20]. A study also showed that high density can lead to leg problems in poultry, with tibial dysplasia and reduced tibial length, width and weight [21]. This change was not observed in the present study. The increase in density in the housed rearing mode limits the space for poultry to move around, and the environment of the paddy field in this study provided the ducks with the space to fulfil their natural habit of swimming and the space to move around, which may be the reason why their growth phenotype was not affected. Moreover, the half-diving length of ducks increased significantly after the increase in stocking density in this study, which may indicate that animal welfare from the ecological stocking model helps to utilize the natural behaviour of ducks.

The liver and spleen indices increased significantly with increasing stocking density in the present study. The liver and spleen are important immune organs in poultry and are closely related to immune system development and immune function. An increase in density in the present experiment study resulted in a significant increase in these two organs, which may reveal adverse health effects. Studies have shown that liver and spleen weights also increase significantly after increasing the stocking density from 14 to 20 birds/m^2^ in broilers [14]. Other studies have also shown an increase in liver and spleen weights with increasing stocking density in broilers and ducks [5,22]. This finding is similar to the results of the present study, where increased stocking density resulted in changes in liver and spleen weights. Increased stocking density may cause a stress response in ducks, which leads to morphological changes in immune organs to promote the immune status of the organism against potential negative effects [23].

In the present study, a significant decrease in serum the ALB concentration was observed after an increase in stocking density. ALB plays a role in the maintenance of plasma osmolality and nutrient metabolism and is mainly synthesized by the liver, reflecting the health of the liver, and a decrease in its level can indicate the degree of liver damage [24]. A study reported that the serum ALB concentration decreased with increasing density at different stocking densities (5, 10 and 15 birds/m^2^) [25]. And studies have shown that a trend of increasing serum CK activity after increasing the stocking density from 15.5 to 20.3 birds/m^2^ and a decreasing ALB concentration [26]. This is consistent with the results of the present study, where increased stocking density resulted in decreased ALB concentration, which suggests that a high stocking density may have a negative impact on liver function. The increased liver indices may also indicate that liver function is affected, resulting in decreased concentrations of ALB produced by the liver. Additionally, the serum CK activity significantly increased in the ducks of in the HD group in this study. The study showed that increased stocking density resulted in increased serum CK activity [27]. This result is consistent with that of the present study. CK is an enzyme involved in energy metabolism, and elevated CK serum activity usually reflects muscle damage or stress [28]. In the RDC coculture system, with increasing stocking density ducks may experience more physical activity and social conflict stress as well as changes in external environmental factors, which may increase serum CK activity.

The small intestinal VH, CD and VH/CD ratio are comprehensive indicators to of the morphology of the small intestine and can reflect the level of intestinal digestion and nutrient utilization [26]. Previous studies have shown that high stocking density has a negative effect on broiler chicken duodenal histomorphology, such as decreasing the VH/CD ratio and increasing the CD [29]. Studies have also reported that a broiler stocking density of up to 40 kg/m^2^ decreased the VH in the duodenum, jejunum and ileum and decreased the jejunal VH/CD ratio [30]. Studies on 1 to 14-day-old goslings showed that the jejunum VH, VH/CD ratio decreased, and the CD of the jejunum and ileum increased at stocking densities up to 30 goslings/m^2^ [15]. The results of the present study showed that the CD of the ileum increased and the VH and VH/CD ratio decreased with increasing density under the RDC coculture system, suggesting that increasing stocking density had a negative effect on the morphology of the ileum in ducks. However, stocking density did not significantly affect the morphology or structure of the duodenum or jejunum in this study, which may be related to the natural resources and environmental activities provided under the ecological farming model, which mitigated the negative effects of increasing density on animal health and welfare by enhancing biological interactions and resource cycling to provide a healthier growing environment for ducks.

The intestinal tract of animals inhabits diverse microbial communities, and the presence of the gut microbiota plays an important role in host health and performance. To investigate the effect of stocking density on the duck gut flora under the RDC coculture system, we analysed the changes in cecal microflora in different groups of ducks using 16S rRNA sequencing, and the results of the diversity index showed that the increase in stocking density did not change the cecal microbial abundance and diversity, which suggests that the increase of density in the RDC system did not cause the change of diversity of cecum microbial colonies in ducks. This was observed in other studies with similar results, where there was no significant difference in the microbial community diversity of the broiler cecum at three caging densities: low (14 birds/m^2^), medium (21 birds/m^2^) and high (28 birds/m^2^) [31]. The structural composition of the microbial community plays an important role in the functioning of the gastrointestinal tract. In the present study, *Bacteroidota* and *Firmicutes* were found to be the dominant phyla in the gut at the phylum level, which is similar to the findings of previous studies [32]. *Firmicutes*, as one of the major bacterial phyla in the gut, is involved in the fermentation of nutrients in the diet and the production of short-chain fatty acids for utilization by the host, which can promote the degradation of polysaccharides and is associated with an enhanced nutrient absorption capacity [33]. *Firmicutes* decreased significantly with increasing stocking density in this experiment. This finding is similar to other studies in which density or group size decreased the abundance of *Firmicutes* [34]. It was also observed in this study that increased stocking density resulted in increased relative abundances of *Deferribacterota* and *Spirochaetota*. *Deferribacterota* were mainly dominated by *Mucispirillum* and *Mucispirillum schaedleri*, and their relative abundances were correlated with plasma inflammatory cytokines and the development of intestinal inflammation [35,36]. *Brachyspira*, a member of *Spirochaetota*, contains anaerobic but oxygen-tolerant spirochetes capable of colonizing the cecum and has been associated with diarrhoea and intestinal inflammation in poultry and mammals [37]. The changes in these flora observed in the present study suggest a possible adverse effect of increased stocking density on duck intestinal health, and we also observed unfavourable changes in ileal morphology in the intestinal morphology. In addition, *Faecalibacterium* is an important genus of short-chain fatty acid-producing bacteria in the animal intestine that inhibits intestinal inflammation, maintains the intestinal barrier and regulates oxidative stress [38]. Low levels of *Faecalibacterium* are usually associated with intestinal inflammation, and the abundance of this bacterium is usually reduced in patients with gastrointestinal disorders [39]. Increased stocking density reduced the relative abundance of *Faecalibacterium* in this study. It was also shown that *Fournierella* plays a role in immunomodulation and maintaining intestinal homeostasis in poultry [40]. However, the increase in density in the present study resulted in a decrease in the abundance of this genus. The results of LEfSe biomarker identification also revealed that some pathogens, such as *Spirochaetota, Proteobacteria, Deferribacteraceae, M. schaedleri*, were markers of the MD and HD groups. *Proteobacteria*, such as *E. coli, Salmonella*, usually cause intestinal diarrhoea in animals [41]. These changes in the flora suggest that increased stocking density affects gut microbial homeostasis and causes intestinal flora dysbiosis.

The PICRUST results showed that increased stocking density reduced the metabolic capacity of microbial genes related to carbohydrate metabolism and amino acid metabolism in the duck cecal flora. Increased stocking density results in dysregulation of gut flora homeostasis, which may be detrimental to adequate metabolism and nutrient utilization, leading to a decrease in the efficiency of energy metabolism. Amino acids are mainly used to promote tissue growth and development while providing energy as metabolic fuel through hepatic gluconeogenesis [42]. The impaired liver function and dysbiosis of the gut flora due to increased density in this study may have combined to affect energy utilization. However, the microbial functional genes were significantly enriched in metabolic pathways such as glycan biosynthesis and metabolism and lipid metabolism. Previous studies have shown that increased stocking density leads to the activation of the body’s energy metabolism to meet energy demands in response to increased density [43]. This different enrichment pathway in the gut microbiota may be related to metabolic changes in gut microbes to adapt to environmental stresses and nutrient competition due to increased density. This was also reflected in the lack of a significant decrease in growth performance. Furthermore, a combination of factors such as nutrient competition, changes in environmental conditions, and microbial interactions and symbiotic relationships under the RDC coculture system may result in differences in changes in the intestinal flora. Subsequent studies need to further analyse the relevant associations or interactions between ecological factors (e.g., soil) and animal gut microorganisms in the RDC coculture systems in rice field environments.

## CONCLUSION

An increase in stocking density under the RDC coculture system did not affect the growth performance or body size traits of young ducks. However, the liver and spleen indices of young ducks increased, serum CK activity increased, and serum ALB concentration decreased at a density of 16 ducks/666.7 m^2^. In addition, increased stocking density adversely affected the morphological structure of the ileum and altered the structure of cecal microbiota, decreasing the abundance of some beneficial bacteria and increasing the abundance of some harmful bacteria. However, this study did not consider the potential impact of the rice field’s ecological environment on the gut microbiota of ducks, which could be further investigated to explore interactions between the environment and gut microbiota and their effects. Additionally, Due to the limited number of replicates in this study, the results can be used as a reference for RDC eco-farming in practical applications, but further validation through larger-scale trials is recommended.

## Figures and Tables

**Table 1:** Diet composition and nutrient levels (as-fed basis)

Items	Content (%)
Ingredient	100.00
Corn	60.20
Soybean meal	18.00
Wheat	15.00
Wheat bran	2.00
Sodium chloride	1.00
Calcium hydrogen phosphate	1.00
Limestone	1.50
DL-Methionine	0.10
L-Lysine HCl	0.20
Premix^1)^	1.00
Nutrient levels^2)^	
Metabolizable Energy (MJ/kg)	12.21
Crude protein (%)	15.07
Calcium (%)	0.93
Available phosphorus (%)	0.32
Total phosphorus (%)	0.58
Methionine (%)	0.34
Lysine (%)	0.85

1) The premix provided per kg of feed: vitamin A 10,000 IU, vitamin D_3_ 3,000 IU, vitamin E 20I U, vitamin B_1_ 1 mg, vitamin B_2_ 4 mg, vitamin B_6_ 1.5 mg, pantothenic acid 10 mg, vitamin B_12_ 0.02 mg, folic acid 1 mg, Niacin 30 mg, biotin 0.15 mg, Cu 8mg, Fe 60 mg, Mn 100mg, Zn 60 mg, I 0.2 mg, Se 0.2 mg.

2) The nutrient levels are calculated values.

**Table 2:** Effects of stocking densities on the growth performance of young ducks in RDC system

Items	Treatment^1)^			SEM	p-value		
	
	LD	MD	HD		ANOVA	Linear	Quadratic
Final weight (g)	983.33	956.67	933.34	13.29	0.096	0.261	0.057
ADG (g/d)	19.00	18.32	17.73	0.32	0.078	0.237	0.046
ADFI (g/d)	89.18	89.18	89.18	0.00	1.000	-	-
F:G (g/g)	4.70	4.87	5.03	0.08	0.067	0.201	0.041

1) LD, 8 birds/666.67 m^2^; MD, 12 birds/666.67 m^2^; HD, 16 birds/666.67 m^2^.

RDC, rice-duck-crayfish; LD, low-density; MD, medium-density; HD, high-density; SEM, standard error of the mean; ANOVA, analysis of variance; ADG, average daily gain; ADFI, average daily feed intake; F: G, feed: gain ratio.

**Table 3:** Effects of different stocking densities on the body size of young ducks in RDC system

Items	Treatment^1)^			SEM	p-value		
	
	LD	MD	HD		ANOVA	Linear	Quadratic
Body oblique length (cm)	12.35	12.95	12.00	0.29	0.091	0.033	0.726
Keel length (cm)	8.80	9.47	9.08	0.29	0.300	0.369	0.205
Tibial length (cm)	6.83	6.86	6.83	0.11	0.969	0.829	0.901
Tibial girth (cm)	3.12	3.08	3.15	0.04	0.571	0.298	1.000
Tibia weight (g)	2.47	2.59	2.57	0.08	0.585	0.862	0.316
Half-diving length (cm)	41.93^b^	42.73^ab^	43.33^a^	0.32	0.023	0.203	0.013

1) LD, 8 birds/666.67 m^2^; MD, 12 birds/666.67 m^2^; HD, 16 birds/666.67 m^2^.

a,b Means with different superscripts in each row differ (p<0.05).

RDC, rice-duck-crayfish; LD, low-density; MD, medium-density; HD, high-density; SEM, standard error of the mean; ANOVA, analysis of variance.

**Table 4:** Effects of stocking densities on the organ indices of young ducks in RDC system

Items (g/kg)	Treatment^1)^			SEM	p-value		
	
	LD	MD	HD		ANOVA	Linear	Quadratic
Heart	6.36	6.74	7.31	0.38	0.245	0.313	0.178
Liver	30.41^b^	34.97^ab^	38.82^a^	1.28	0.001	0.049	0.001
Spleen	0.48^b^	0.64^ab^	0.72^a^	0.05	0.012	0.261	0.005
Pancreas	3.51	4.15	3.94	0.22	0.147	0.496	0.067
Gizzard	44.98	49.57	47.08	1.47	0.122	0.251	0.083
Proventriculus	4.03	4.29	4.31	0.18	0.499	0.918	0.248

1) LD, 8 birds/666.67 m^2^; MD, 12 birds/666.67 m^2^; HD, 16 birds/666.67 m^2^.

a,b Means with different superscripts in each row differ (p<0.05).

RDC, rice-duck-crayfish; LD, low-density; MD, medium-density; HD, high-density; SEM, standard error of the mean; ANOVA, analysis of variance.

**Table 5:** Effects of stocking densities on the serum biochemistry of young ducks in RDC system

Items	Treatment^1)^			SEM	p-value		
	
	LD	MD	HD		ANOVA	Linear	Quadratic
TBIL (μmol/L)	36.31	45.02	51.34	4.41	0.084	0.327	0.044
TP (g/L)	46.87	43.56	41.36	1.56	0.072	0.336	0.036
ALB (g/L)	25.21^a^	23.09^ab^	20.88^b^	1.00	0.027	0.139	0.019
ALP (U/L)	581.18	620.57	484.91	59.75	0.286	0.129	0.703
ALT (U/L)	50.40	39.32	49.55	4.85	0.231	0.157	0.331
AST (U/L)	51.36	36.47	39.74	6.99	0.314	0.745	0.142
TG (mmol/L)	1.14	1.52	1.42	0.14	0.17	0.613	0.073
TC (mmol/L)	6.23	5.22	5.21	0.56	0.357	0.984	0.158
GLU (mmol/L)	10.71	9.36	9.54	0.50	0.145	0.797	0.055
CK (U/L)	1,008.93^b^	1,076.34^ab^	1,202.90^a^	47.50	0.033	0.079	0.040
HDL (mmol/L)	3.71	3.28	3.36	0.35	0.664	0.863	0.382
LDL (mmol/L)	1.81	1.25	1.20	0.33	0.358	0.905	0.160
IgG (g/L)	0.62	0.76	0.72	0.05	0.149	0.515	0.067
CHE (U/L)	4,220.05	5,057.07	3,627.99	410.38	0.077	0.026	0.811
GLB (g/L)	21.66	20.47	20.81	0.91	0.640	0.791	0.372
A/G	1.17	1.14	1.01	0.05	0.097	0.101	0.142

1) LD, 8 birds/666.67 m^2^; MD, 12 birds/666.67 m^2^; HD, 16 birds/666.67 m^2^.

a,b Means with different superscripts in each row differ (p<0.05).

RDC, rice-duck-crayfish; LD, low-density; MD, medium-density; HD, high-density; SEM, standard error of the mean; ANOVA, analysis of variance; TBIL, total bilirubin; TP, total protein; ALB, albumin; ALP, alkaline phosphatase; ALT, alanine aminotransferase; AST, aspartate aminotransferase; TG, triglyceride; TC, total cholesterol; GLU, glucose; CK, creatine kinase; HDL, high-density lipoprotein; LDL, low-density lipoprotein; IgG, immunoglobulin G; CHE, cholinesterase; GLB, globulin; A/G, albumin/globulin ratio.

**Table 6:** Effects of stocking densities on the intestinal morphology of young ducks in RDC system

Items	Treatment^1)^			SEM	p-value		
	
	LD	MD	HD		ANOVA	Linear	Quadratic
Duodenum							
VH (μm)	1,068.43	1,077.16	1,122.80	11.97	0.137	0.064	0.462
CD (μm)	192.55	201.69	201.94	3.69	0.508	0.310	0.572
VH/CD	5.83	5.48	5.64	0.15	0.657	0.640	0.432
Jejunum							
VH (μm)	893.10	861.46	866.46	12.83	0.721	0.420	0.987
CD (μm)	179.95	176.67	195.83	4.25	0.344	0.151	0.801
VH/CD	5.15	5.02	4.60	0.13	0.228	0.088	0.872
Ileum							
VH (μm)	738.14^a^	727.34^ab^	699.30^b^	6.66	0.045	0.016	0.538
CD (μm)	186.77^b^	220.69^a^	216.07^a^	4.46	0.002	0.004	0.035
VH/CD	4.04^a^	3.41^b^	3.33^b^	0.08	0.001	0.001	0.100

1) LD, 8 birds/666.67 m^2^; MD, 12 birds/666.67 m^2^; HD, 16 birds/666.67 m^2^.

a,b Means with different superscripts in each row differ (p<0.05).

RDC, rice-duck-crayfish; LD, low-density; MD, medium-density; HD, high-density; SEM, standard error of the mean; ANOVA, analysis of variance; VH, villus height; CD, crypt depth; VH/CD, villus height/crypt depth.
